# Buffered aspirin: what is your gut feeling?

**DOI:** 10.1007/s12471-014-0516-1

**Published:** 2014-01-31

**Authors:** B. C. du Pré, L. W. van Laake

**Affiliations:** 1 Department of Cardiology, University Medical Center Utrecht, Heidelberglaan 100, 3584 CX Utrecht, the Netherlands; 2 Department of Cardiology and Medical Physiology, University Medical Center Utrecht, Utrecht, the Netherlands

Aspirin (acetylsalicylic acid, ASA) is one of the oldest and most commonly used drugs in the world. In the 5th century BC, Hippocrates already prescribed leaves and bark of the willow tree, consisting of ASA precursor salicin, to patients with high fever and extreme pains. Nowadays, chronic low-dose ASA is widely prescribed as a platelet aggregation inhibitor in patients at risk for myocardial infarction or stroke. In the Netherlands, it is the second most used drug with 7.6 million prescriptions in 2012 [1]. One of the main disadvantages of ASA, however, is up-regulation of COX-2 in the stomach, which may result in gastrointestinal side effects including dyspepsia, gastrointestinal ulcers, and bleeding [2]. These side effects instigated the development of alternatives such as effervescent carbasalate calcium (ECC). ECC is a salt formulation of ASA, which makes it more soluble and theoretically prevents contact of high drug concentrations with the gastric mucosa. Initial animal and clinical studies in volunteers with high-dose drug administration indeed showed a reduced incidence of gastrointestinal side effects in ECC users [3, 4]. Later, however, patient studies with low-dose drugs showed that the risk of ulcer development and bleeding is similar for ECC and ASA [5, 6].

In the current issue of the Netherlands Heart Journal, Focks et al. [7] describe that in addition to ulcer development and bleeding, the risk of self-reported gastrointestinal symptoms of ECC and ASA is also comparable. In their observational study using questionnaires, gastrointestinal symptoms were similar in patients receiving ECC and ASA. As the authors acknowledge in the discussion, the study design involves the risk of response bias and confounding by indication. Although the authors corrected for potential confounders, it might still be possible that response bias and confounding mask a small advantage of ECC. In combination with previous studies comparing ECC and ASA on ulcer development, however, this study supports the hypothesis that gastrointestinal side effects of ASA are mainly attributed to the systemic presence of ASA rather than the local effect on the gastric mucosa [5].

This article is a fine example of a phase 5 study; a relatively new term referring to studies that investigate effectiveness and side effects in a community after a therapy has proven to work in large clinical trials [8, 9]. Conditions such as comorbidities, decision-making of the practitioner, and patient compliance, which are excluded or minimised in the previous phases but play an important role in daily practice, are taken into account. The first four study phases investigate whether a new therapy works in *defined* –and often highly selected patient groups; phase 5 analyses whether a *community* benefits from the new therapy *in daily practice*. Although phase 5 studies are essential, they are infrequently conducted. Companies invest a lot of money in new therapies and do not want to risk negative consequences once a therapy is on the market. Researchers may be less interested because the appreciation of phase 5 studies is generally lower due to the observational study design and its inherent risk of bias, which will likely be emphasised by colleagues and the company selling the therapy.

The impact of this kind of research is nonetheless large. As Focks et al. point out, ECC costs twice as much as ASA. In the Netherlands, this results in 5 million euros annually (on top of ASA costs) for a therapy, which in multiple studies shows no additional beneficial effects. Focks and colleagues nicely demonstrated that although ECC works better in theory, in pre-clinical studies, and even in some clinical studies, in daily practice it is not superior to the cheaper ASA. In an era with loads of new, expensive therapies and more cost-awareness, community-based effectiveness studies are vital to gain maximum profit out of the available healthcare budget.
